# HBV‐Induced Carcinogenesis: Mechanisms, Correlation With Viral Suppression, and Implications for Treatment

**DOI:** 10.1111/liv.16202

**Published:** 2024-12-25

**Authors:** Thomas Tu, Thomas J. McQuaid, Ira M. Jacobson

**Affiliations:** ^1^ Storr Liver Centre, Westmead Clinical School, Centre for Infectious Diseases and Microbiology and Westmead Institute for Medical Research The University of Sydney Sydney New South Wales Australia; ^2^ Gilead Sciences Inc. Provincetown Massachusetts USA; ^3^ NYU Langone Health New York New York USA

**Keywords:** antiviral therapy, chronic hepatitis B virus, HBV infection, hepatocellular carcinoma

## Abstract

**Background:**

Chronic hepatitis B virus (HBV) infection is a common but underdiagnosed and undertreated health condition and is the leading cause of hepatocellular carcinoma (HCC) worldwide. HBV (rated a Grade 1 carcinogen by the International Agency for Research on Cancer) drives the transformation of hepatocytes in multiple ways by inducing viral DNA integrations, genetic dysregulation, chromosomal translocations, chronic inflammation, and oncogenic pathways facilitated by some HBV proteins. Importantly, these mechanisms are active throughout all phases of HBV infection. Nevertheless, most clinical guidelines for antiviral therapy recommend treatment based on a complex combination of HBV DNA levels, transaminasemia, liver histology, and demographic factors, rather than prompt treatment for all people with infection.

**Aims:**

To determine if current frameworks for antiviral treatment address the impacts of chronic HBV infection particularly preventing cancer development.

**Materials and Methods:**

We reviewed the recent data demonstrating pro‐oncogenic factors acting throughout a chronic HBV infection can be inhibited by antiviral therapy.

**Results:**

We extensively reviewed Hepatitis B virology data and correlating clinical outcome data. From thi, we suggest that new findings support simplifying and expanding treatment initiation to reduce the incidence ofnew infections, progressive liver disease, and risk of hepatocellular carcinoma. We also consider lessons learned from other blood‐borne pathogens, including the benefits of antiviral treatment in preventing transmission, reducing stigma, and reframing treatment as cancer prevention.

**Conclusion:**

Incorporating these practice changes into treatment is likely to reduce the overall burden of chronic HBV infections and HCC. Through this, we may better achieve the World Health Organization's goal of eliminating viral hepatitis as a public health threat and minimise its impact on people's lives.

**Summary**

- Chronic hepatitis B virus (HBV) infection is a common but underdiagnosed and undertreated health condition and is the leading cause of hepatocellular carcinoma worldwide.
- Recent advances in the study of HBV biology have highlighted the potential for early intracellular events in the lifetime of infected people, including viral integration and viral protein expression, to lead to hepatocellular carcinoma many years later.
- The events that culminate in hepatocarcinogenesis are amenable to intervention with antiviral therapy. Existing guidelines for treatment do not advise prompt antiviral therapy but instead recommend complex criteria for reactive therapy.
- Universal treatment of HBV has been demonstrated to be cost‐effective in most of the developed world.
- The case for offering nucleotide reverse transcriptase inhibitors to a broader spectrum of viremic patients with chronic hepatitis B to prevent cancer is increasingly compelling.

**Abbreviations**
AASLD: American Association for the Study of Liver Diseases
AE: adverse event
AFP: alpha‐fetoprotein
ALT: alanine aminotransferase
APASL: Asian Pacific Association for the Study of the Liver
cccDNA: covalently closed circular DNA
CHB: chronic hepatitis B
CNA: copy number alterations
dslDNA: double‐stranded linear DNA
EASL: European Association for the Study of the Liver
HBcrAg: hepatitis B core‐related antigen
HBeAg: hepatitis B e antigen
HBsAg: hepatitis B surface antigen
HBV: hepatitis B virus
HBx: hepatitis viral protein
HCC: hepatocellular carcinoma
HIV: human immunodeficiency virus
JNK: c‐Jun N‐terminal kinase
NA: nucleos(t)ide analog
PI3K: phosphoinositide‐3‐kinase
SMC: structural maintenance of chromosomes
TAF: tenofovir alafenamide
TDF: tenofovir disoproxil fumarate
TERT: telomerase reverse transcriptase
ULN: upper limit of normal
UPS: ubiquitin proteasome system

## 1 Introduction

Worldwide, approximately 296 million people are chronically infected with the hepatitis B virus (HBV). Yet, it's estimated only 10% of patients are aware of their infection [1]. Low diagnosis rates are not limited to lower‐resource nations; in the US, only 15% of people with HBV know their status, and only 5% of all HBV‐infected persons receive antiviral therapy [2]. HBV transmission occurs via exposure to body fluids and can occur either horizontally or vertically [3, 4, 5]. In the absence of effective vaccination (many patient subgroups do not respond well to standard vaccination), infection in infancy or early childhood results in chronic active infection and hepatitis 95% of the time, vs. 5% to as high as 28% if infection occurs in adulthood [1, 6].

Chronic infection with HBV causes over 800 000 deaths each year, primarily from complications secondary to liver cirrhosis and hepatocellular carcinoma (HCC) [1, 7, 8, 9]. While not all people with chronic HBV infection develop HCC, high viral replication is associated with significantly increased lifetime cancer risk [10].

**FIGURE 1:**
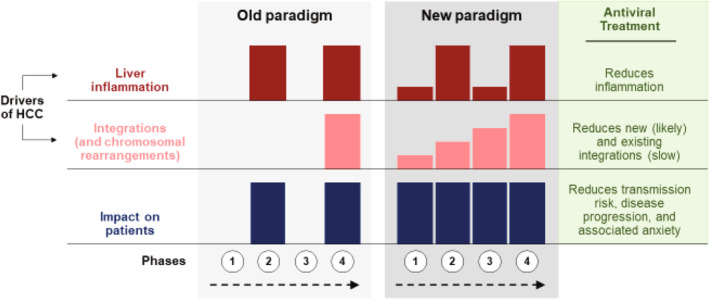
Old vs. new paradigm of the stages of chronic HBV infection. Phases may be referred to as follows: (1) HBeAg‐positive chronic infection or “immune tolerant”; (2) HBeAg‐positive chronic hepatitis or “immune active”; (3) HBeAg‐negative chronic infection or “inactive carrier”; and (4) HBeAg‐negative chronic hepatitis or “reactivation.” HBeAg, hepatitis B e antigen; HBV, hepatitis B virus; HCC, hepatocellular carcinoma.

Multiple pathways lead to the development of HCC, and these (potentially synergistic [11, 12]) pathogenic processes can occur throughout chronic infection [13]. Until recently, it was thought that HBV infection early in life could exist for decades without initiating an inflammatory response, historically referred to as the “immune‐tolerant” phase. However, studies have shown the limited ability of ALT levels to predict the absence of intrahepatic inflammation and, in particular, HBV‐specific T cell activity (Table 1 and Figure 1) [14, 15, 16, 17]. In a particularly impactful study, Mason et al. [14] demonstrated not only histologic inflammatory activity in a cohort of “immune‐tolerant” patients, but also peripheral and hepatic HBV‐specific T‐cell activity and clonal expansion of hepatocytes to a degree greater than expected by mathematical modelling. Bertoletti and Kennedy [18] subsequently expanded upon the concept of greater proactivity regarding therapy by highlighting the evidence that young “immune‐tolerant” patients do have an immunologic response to HBV even if it does not result in the levels of inflammation needed to raise ALT levels. These and subsequent authors have noted the potential oncogenic implications of clonal expansion in the form of reservoirs of cells that may ultimately result in dysplasia or neoplasia [19]. Moreover, integration of viral DNA into hepatocyte chromosomes has been shown to occur early and throughout infection in the presence of active viral replication, with the potential for insertional mutagenesis and chromosomal translocations [20, 21]. These mechanisms at play early in infection raise several important issues, in particular, the need for considering changes in the current treatment paradigm.

**TABLE 1:** Natural history and chronic states of HBV per AASLD and EASL guidelines [4, 7].

Older terminology used by AASLD^a^	Immune tolerant	Immune active	Low replication	Reactivation	Clearance
More accurately described as^b^	HBeAg‐positive chronic HBV infection	HBeAg‐positive chronic hepatitis B	HBeAg‐negative chronic HBV infection	HBeAg‐negative chronic hepatitis B	Negative
					HBsAg and positive antibodies to HBcAg
Level of inflammation	Minimal	Moderate/severe	Minimal	Active	Minimal
Fibrosis	None	With or without	Variable levels	Present	With or without
ALT	Normal/elevated	Elevated	Normal	Elevated^c^	Normal
HBV DNA (IU/mL)	> 10^7^	10^4^–10^7^	< 2000	> 2000^d^	Usually undetectable
HBsAg	High	High/intermediate	Low	Intermediate	Negative
HBeAg	Positive	Positive	Negative	Negative	

Abbreviations: AASLD, American Association for the Study of Liver Diseases; ALT, alanine aminotransferase; EASL, European Association for the Study of the Liver; HBcAg, HBV core antigen; HBeAg, hepatitis B e antigen; HBsAg, hepatitis B surface antigen; HBV, hepatitis B virus.

^a^ Phase terminology from the AASLD guidelines.

^b^ Alternative phrase terminology per the EASL clinical practice guidelines.

^c^ Persistently or intermittently.

^d^ Higher than baseline.

**TABLE 2:** HBV antiviral treatment guidelines aimed at improving survival, reducing disease progression, and reducing the incidence of HCC.

	Initial assessment	Who should be treated	Treatment
Chinese Guidelines for Prevention and Treatment of CHB [22]	Patients should be assessed to determine whether to start antiviral therapy based on comprehensive analysis of serum HBV DNA levels, ALT levels, and the severity of liver disease, as well as their age, family history, and concomitant diseases	For patients with CHB infection positive for serum HBV DNA, antiviral therapy is indicated if ALT levels are persistently abnormal (> ULN) and other causes of ALT elevation have been excluded If patients are seropositive for HBV DNA with normal ALT levels, antiviral therapy is indicated if meeting any one of the following criteria: (1) liver biopsy suggesting obvious inflammation (≥ G2), fibrosis (≥ S2), or both; (2) family history of HBV‐related cirrhosis or liver cancer and age > 30 years; (3) noninvasive tests or liver biopsy revealing obvious liver inflammation or fibrosis in those with persistently normal ALT and age > 30 years, without family history of cirrhosis or liver cancer; and (4) HBV‐related extrahepatic manifestations	HBeAg‐positive or ‐negative CHB patients should be treated with ETV, TDF, or TAF HBeAg‐positive or ‐negative CHB patients could be treated with peg‐IFN‐α initially For patients with HBV‐related compensated cirrhosis, long‐term treatment with ETV, TDF, or TAF, or peg‐IFN‐α therapy is recommended
US Treatment Algorithm [23]	Not included in guideline	All patients (HBeAg‐positive or ‐negative) with HBV DNA ≥ 2000 IU/mL and elevated ALT (> 35 IU/mL for men and > 25 IU/mL for women) If patients with HBV DNA ≥ 2000 IU/mL and elevated ALT without fibrosis are not treated, their HBV DNA and ALT levels should be monitored every 3–6 months	Peg‐IFN alfa‐2a, ETV, TDF, and TAF are first‐line therapies HBeAg‐positive patients with evidence of less extensive fibrosis (< F3) should be treated long‐term For HBeAg‐negative patients without HBsAg seroconversion, the panel does not recommend stopping treatment
AASLD [4]	Screening should be performed using both HBsAg and anti‐HBs Screening is recommended in all those born in countries with HBsAg seroprevalence ≥ 2%, US‐born persons not vaccinated as infants with parents from regions with high HBV endemicity (≥ 8%), pregnant women, persons needing immunosuppressive therapy, and other groups at high risk Anti‐HBs–negative screened persons should be vaccinated Screening for anti‐HBc to check prior exposure is not routinely recommended but is important in those with HIV, those about to undergo HCV or anticancer and other immunosuppressive therapies or renal dialysis, and in donated blood (or, if feasible, organs)	Antiviral therapy is recommended for adults with immune‐active CHB (HBeAg negative or positive) to decrease the risk of liver‐related complications Immune‐active CHB is defined by an elevation of ALT ≥ 2 ULN or evidence of significant histologic disease plus HBV DNA > 2000 IU/mL (HBeAg negative) or > 20 000 IU/mL (HBeAg positive) Antiviral therapy for adults with immune‐tolerant CHB is not recommended	Peg‐IFN, ETV, and TDF are preferred initial therapies for adults with immune‐active CHB Antiviral therapy is suggested to be indefinite for HBeAg‐positive adults with cirrhosis with CHB who seroconvert to anti‐HBe on NA therapy, based on concerns for potential clinical decompensation and death, unless there is a strong competing rationale for treatment discontinuation Antiviral therapy is suggested for adults with HBeAg‐negative, immune‐active CHB unless there is a compelling rationale for treatment discontinuation Persons with persistent low‐level viremia (< 2000 IU/mL) on ETV or tenofovir monotherapy should continue monotherapy regardless of ALT level
APASL [24]	The following groups should be tested for HBV infection: ○ Persons with liver disease ○ Persons needing immunosuppressive or cancer chemotherapy ○ Injection drug users ○ Persons who have received unsafe injections (used syringes or needles) ○ Men who have sex with men ○ Persons with multiple sexual partners or a history of sexually transmitted infection ○ Family members, household contacts, and sex partners of a person with HBV ○ Inmates of correctional facilities ○ Dialysis patients ○ HCV‐ or HIV‐infected individuals ○ Pregnant females (preferably during the first trimester to vaccinate unprotected mothers) ○ Infants born to females with CHB ○ Blood or organ donors ○ Health care workers	The initial evaluation of an individual with HBV infection should include assessment of the level of viremia, degree of inflammation, and presence and stage of liver disease Patients with decompensated cirrhosis and detectable HBV DNA require urgent antiviral treatment with NA(s) Patients with compensated cirrhosis and HBV DNA (2000 IU/mL) should also be considered for treatment even if ALT levels are normal Treatment may be started in precirrhotic patients with CHB if they have persistently elevated ALT (2× ULN, at least 1 month between observations) and HBV DNA levels (20 000 IU/mL if HBeAg‐positive and 2000 IU/mL if HBeAg‐negative)	Treatment‐naïve patients can be treated with TDF, ETV, ADV, LDT, or LAM; TDF and ETV are the preferred NAs and should be used as first‐line therapies During NA therapy, HBeAg, anti‐HBe (in patients who are HBeAg positive), and ALT levels should be monitored every 3 months The optimal duration of NA therapy is unknown in patients with HBeAg‐negative CHB. In patients without liver cirrhosis, the treatment can be withdrawn (1) after HBsAg loss following either anti‐HBs seroconversion or at least 12 months of a post–HBsAg clearance consolidation period, or (2) after treatment for at least 2 years with undetectable HBV DNA documented on 3 separate occasions, 6 months apart
EASL [7]	Initial evaluation of a subject with CHB infection should include a complete history, a physical examination, and assessment of liver disease activity, severity, and markers of HBV infection HBeAg and anti‐HBe detection are essential for the determination of the phase of CHB infection	All patients with HBeAg‐positive or ‐negative CHB, defined by HBV DNA 2000 IU/mL, ALT ULN, and/or at least moderate liver necroinflammation or fibrosis, should be treated; patients with compensated or decompensated cirrhosis need treatment, with any detectable HBV DNA level and regardless of ALT levels; patients with HBV DNA 20 000 IU/mL and ALT 2× ULN; patients with HBeAg‐positive CHB infection, defined by persistently normal ALT and high HBV DNA levels, who are older than 30 years; patients with HBeAg‐positive or HBeAg‐negative CHB infection and family history of HCC or cirrhosis and extrahepatic manifestations	The long‐term administration of a potent NA with high barrier to resistance is the treatment of choice regardless of the severity of liver disease The preferred regimens are ETV, TDF, and TAF as monotherapies LAM, ADV, and TBV are not recommended in the treatment of CHB NAs should be discontinued after confirmed HBsAg loss, with or without anti‐HBs seroconversion Patients under effective long‐term NA therapy should remain under surveillance for HCC Peg‐IFN‐α can be considered as an initial treatment option for patients with mild to moderate HBeAg‐positive or ‐negative CHB

Abbreviations: AASLD, American Association for the Study of Liver Diseases; ADV, adefovir; ALT, alanine aminotransferase; APASL, Asian Pacific Association for the Study of the Liver; CHB, chronic hepatitis B; EASL, European Association for the Study of the Liver; ETV, entecavir; HBeAg, hepatitis B e antigen; HBsAg, hepatitis B surface antigen; HBV, hepatitis B virus; HCC, hepatocellular carcinoma; HCV, hepatitis C virus; HIV, human immunodeficiency virus; LAM, lamivudine; LDT, telbivudine; NA, nucleos(t)ide analog; Peg‐IFN‐α, pegylated interferon alfa‐2a; TAF, tenofovir alafenamide; TDF, tenofovir disoproxil fumarate; ULN, upper limit of normal.

**TABLE 3:** Evidence for the reduction of HCC risk by antiviral treatment.

Author (year)	Population	Antiviral	Follow‐up	Treatment effect vs. control
Kim et al. (2015) [25]	634 patients, 482 without cirrhosis (76%) and 152 patients (24%) with cirrhosis	TDF	384 weeks	Without cirrhosis: SIR, 0.40 (95% CI, 0.20–0.80); with cirrhosis: SIR, 0.51 (95% CI, 0.23–1.14)
Ahn et al. (2016) [26]	646 (9.4% with cirrhosis)	ETV	Maximum follow‐up time of 8.2 years	SIR, 0.56 (95% CI, 0.35–0.905); 17 cases vs. expected 30.2 cases overall; without cirrhosis: SIR, 0.37 (95% CI, 0.166–0.82)
Hoang et al. (2016) [27]	Total 3665 patients, without cirrhosis: untreated patients from REVEAL‐HBV (aged 30–65 years from Taiwan), and treated or untreated patients from Northern California	Any of the FDA‐approved agents: LAM, ADV, ETV, LDT, tenofovir, interferon, or combinations	Overall median follow‐up time was 8.9 years	US‐treated group compared to the combined US‐REVEAL‐untreated group: adjusted HR, 0.31 (95% CI, 0.14–0.67); *p* = 0.0027
Lin et al. (2016) [28]	2255 US patients, 3653 Taiwanese patients from REVEAL‐HBV	ETV (46.9%); tenofovir (22.2%); ADV (19%); LAM (10.5%); LDT (0.8%); emtricitabine (0.5%); Peg‐IFN (0.1%)	5840 person‐years (US cohort); 57 499 person‐years (Taiwan cohort)	US cohort, treated vs. untreated: HR, 0.31 (95% CI, 0.15–0.66); *p* = 0.002; treated US vs. Taiwan cohort: HR, 0.22 (95% CI, 0.12–0.40); *p* < 0.001
Su et al. (2016) [29]	1315 ETV‐treated and 503 untreated patients with cirrhosis	ETV	Median treatment and follow‐up durations of 4 and 6 years, respectively	60% HCC risk reduction: HR, 0.40 (95% CI, 0.28–0.57)
Kim et al. (2018) [30]	IT group (*n* = 413), IA group treated with NA (*n* = 1497), and MA group (*n* = 1141)	NA	6.3 years (IT and IA groups)	HCC in the IT vs. IA groups was 4.2% vs. 1.6% at 5 years and 12.7% vs. 6.1% at 10 years (*p* = 0.001); MA vs. IA: HR 3.23; 95% CI 2.28–4.57; *p* < 0.001
Liu et al. (2019) [31]	1088 (291 untreated and 797 treated) CHB patients with cirrhosis	TDF	5‐year cumulative probability	HCC rate of 14.9% vs. 9.8% for untreated and treated, respectively (*p* = 0.07)
Jiang (2021) [32]	362 patients with CHB and 96 with HBV cirrhosis without antiviral treatment; 203 CHB and 129 HBV cirrhosis patients receiving antiviral therapy	Antiviral group: Monotherapy with LAM 100 mg/day, ADV 10 mg/day, LDT 600 mg/day, or ETV 0.5 mg/day; combination therapy consisted of an initial combination or salvage treatment, namely, LAM + ADV, LDT + ADV, or ETV + ADV	Median follow‐up time was 10 years (antiviral group); in the control group, median follow‐up time was 8 years	Amongst CHB and HBV cirrhosis patients, cumulative incidence rates of HCC were 14.9% and 53.1%, respectively, without antivirals vs. 10.7% and 31.9%, respectively, with antivirals
Li et al. (2022) [33]	315 CHB patients were enrolled and divided into NA (*n* = 144) and non‐NA (*n* = 171)	NA		HCC and mortality in the NA group were significantly lower vs. the non‐NA group

Abbreviations: ADV, adefovir; CHB, chronic hepatitis B; CI, confidence interval; ETV, entecavir; FDA, Food and Drug Administration; HCC, hepatocellular carcinoma; HR, hazard ratio; IA, immune active; IT, immune tolerant; LAM, lamivudine; LDT, telbivudine; MA, mildly active; NA, nucleos(t)ide analog; SIR, standardised incidence rate; TAF, tenofovir alafenamide; TDF, tenofovir disoproxil fumarate.

The goals of treatment, as indicated in major international guidelines (Table 2), include improving survival and quality of life, preventing disease progression (e.g., cirrhosis, decompensation), and reducing the incidence of liver transplantation and HCC [4, 7]. Table 3 summarises clinical trials showing the efficacy of antiviral treatment vs. no antiviral treatment for preventing the development of HCC in patients with HBV. Several studies have shown reductions in HCC risk varying from 60% to nearly 80% with antiviral therapy vs. no treatment [28, 29]. These considerations led Zoulim and Mason [34], as early as 2012, to make a plea for earlier antiviral therapy in light of the potency and the high barrier to resistance of the then, newer antiviral agents, tenofovir, and entecavir.

Inflammation represents an ongoing risk for cancer and is alleviated with antiviral treatment. The pathogenic inflammation associated with chronic HBV infection is often driven by the recruitment of inflammatory monocytes, T, and NK cells into the liver, leading to non‐specific hepatocyte killing, whereas effective HBV‐specific immunity driven by T cells can occur in the absence of pathohistological changes or increases in ALTs [35, 36]. Increasing evidence suggests that antiviral treatment reduces the former while restoring the latter [35]. Moreover, although liver inflammation and cirrhosis are important factors driving the development of HCC, approximately 10%–20% of HCC cases occur in their absence [37, 38].

Despite these findings, the guidelines of prevailing professional societies (AASLD, EASL, and APASL) still base their treatment recommendations on defined levels of HBV DNA, ALT, and histological abnormalities including inflammation and fibrosis. Here, we describe potentially procarcinogenic mechanisms that occur throughout the course of HBV infection. Informed by these descriptions, we will comment on the ability of nucleoside treatment to prevent HBV‐associated HCC and thus make the case for expanding the current recommendations for treatment initiation.

## 2 Mechanisms of Carcinogenesis

Malignant transformation by HBV is driven by a combination of the potentially oncogenic properties of virally expressed proteins, genetic alterations driven by viral DNA host‐cell integrations, and chronic inflammation and its role in driving cell turnover and thus clonal expansion.

### 2.1 Hepatitis B Viral Proteins

HBx is a nonstructural transactivating protein that is necessary for the establishment of HBV infection in host hepatocytes and can affect the stability of host DNA [39]. HBx mediates the transcriptional activity of cccDNA [40] by degrading structural maintenance of chromosomes (SMC) complex proteins that inhibit HBV gene expression [41]. HBx also has a complicated set of interactions with the ubiquitin proteasome system (UPS). While the UPS can degrade viral proteins such as HBx, HBx can use different components of the UPS to promote HCC development via the ubiquitination of proteins mediated by the UPS to regulate cellular proliferation and other processes [42]. The increased viral transcription that follows protein degradation may have implications for transcriptional activation, notably in regions of host‐cell chromosomes that are involved in cell cycle regulation or cell differentiation. The SMC complex has diverse properties that contribute to genomic stability, including the formation of chromatin loops and topologically associating domains [43], binding to double‐stranded DNA [44], and formation of gamma‐H2Ax [45]. HBx‐mediated degradation of the SMC complex can therefore result in genomic instability [41].

Thus, HBx has been reported to potentiate HCC development [46, 47, 48]. In the context of its pluripotential biologic properties, HBx has been reported to induce hepatocytes to enter and remain in the G1 phase of the cell cycle [49]; additionally, the appearance of hepatic adenomas and progression to HCC occur in animal models that overexpress HBx [50], possibly through impairing DNA double‐stranded break repair [51]. Truncated variants of HBx (including C‐terminal–truncated HBx created by random HBV genome integration) can also confer enhanced invasiveness and lessen apoptotic response in HCC cells [52]. Downstream pathways, such as the PI3K/AKT signalling pathway, can be activated by HBx overexpression. HBx drives expression of alpha‐fetoprotein (AFP), which is associated with 70%–90% of patients with hepatocyte tumours; it can activate the PI3K/AKT signalling pathway to promote the transformation of liver cells into cancer [53, 54]. HBx, along with transforming growth factor beta 1, may induce the transformation of hepatic progenitor cells into hepatic cancer stem cells via the c‐Jun N‐terminal kinase (JNK)/c‐Jun pathway [55]. While HBx has been associated with oncogenic‐related cellular pathways, the understanding of its specific role in tumorigenesis has been hampered by its involvement in numerous cellular activities and the wide variation in the amount of HBx seen depending on HBV viral load, tumour microenvironment, and other risk factors involved [56]. Nucleos(t)ide analog (NA) antiviral therapy has been associated with the development of HBx mutants with decreased HBV replication efficiency, suggesting a potential benefit of early NA administration even in the absence of noticeable inflammation or fibrosis [57].

Mutations in the pre‐S2 domain of hepatitis B surface antigen (HBsAg) are also associated with HCC [58]. A cross‐sectional study of HBsAg‐positive patients showed that patients with HCC had a higher prevalence of pre‐S1 and/or pre‐S2 deletions compared to non‐HCC patients [58]. In a separate cohort of Italian patients, investigators found that pre‐S2 mutations were detected in about half of HBV patients, with their presence in serum HBV DNA significantly associated with active infection, liver disease, and HCC prevalence [59].

Thus, constitutive expression of viral proteins during chronic infection may drive carcinogenesis. Lowering the expression of viral proteins will likely reduce tumour development; multiple antiviral compounds (including tranilast, domiphen bromide, azithromycin, alexidine hydrochloride, ammonium glycyrrhizinate, and valsartan) have been shown to possess HBx‐binding activity, which could be expected to aid in preventing tumorigenesis [60], in addition to potential HBx‐related effects of NA on viral replication.

### 2.2 HBV DNA Integrations and Their Role in HCC Development

In HBV infection, mispriming during viral replication can result in double‐stranded linear DNA (dslDNA) fragments, which integrate into the hepatocyte genome. These integrants persist in the hepatocyte genome and can act as stable transcriptional templates of the procarcinogenic viral proteins HBx and HBsAg. The HBx protein is expressed by these integrated HBV DNA templates as well as by HBV cccDNA [61]. Recent evidence indicates that cccDNA is lost during mitosis and is not present in daughter cells [62]. These data further support the importance of considering earlier initiation of antiviral treatment than is typically recommended in published guidances or guidelines. Thus, the case could be made that earlier treatment results in the expansion of the population of uninfected hepatocytes over the long term. These HBV‐mediated mutagenic pathways help to explain why, in chronic HBV (CHB), HCC may develop in the absence of fibrosis, cirrhosis, and transaminasemia [63, 64, 65].

**FIGURE 2:**
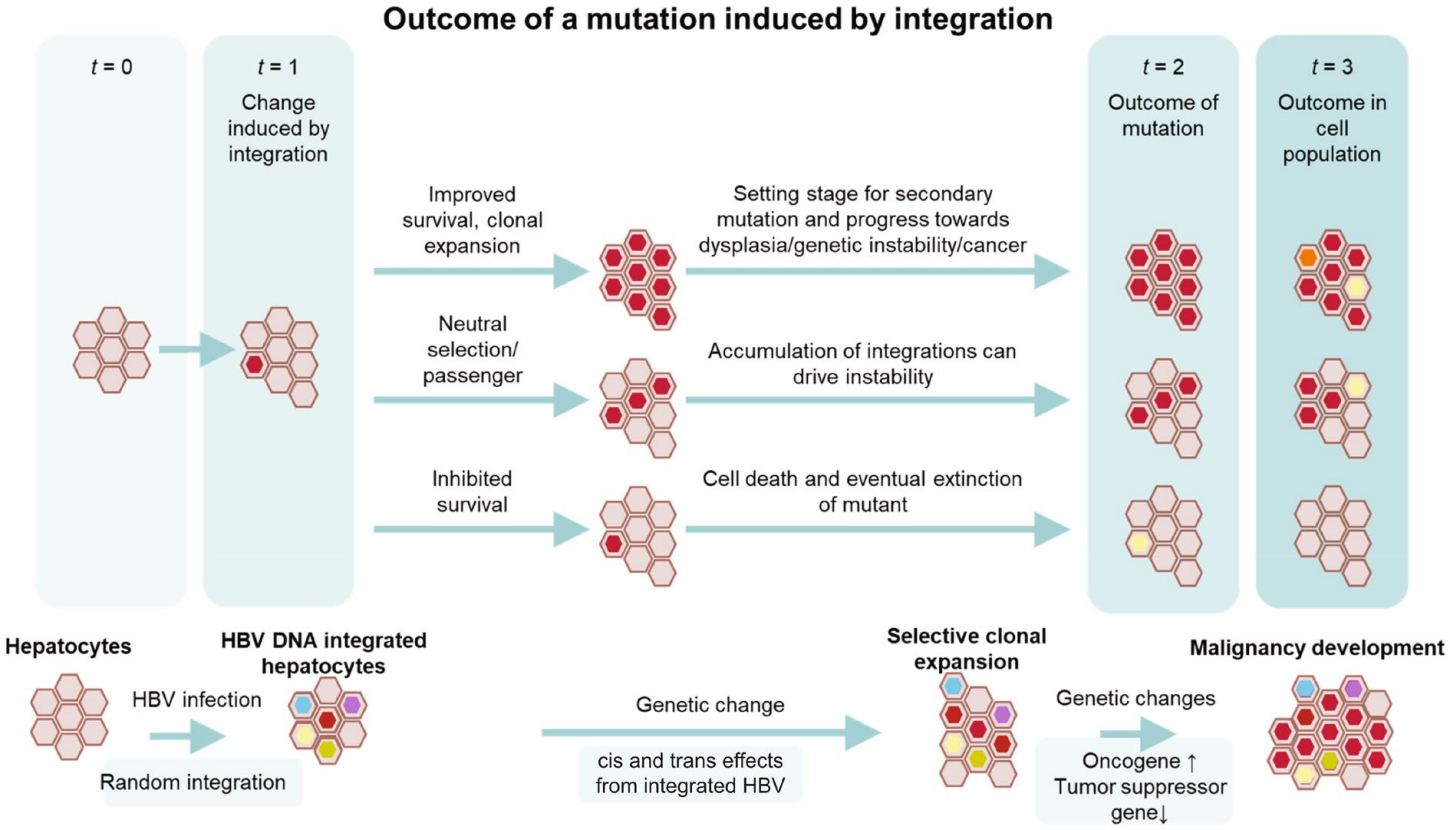
Relationship of HBV DNA integration to HCC development [79]. It is often the case that a mutation is either lethal to the cell or results in a survival disadvantage for the cell. In this case, the mutant cell will disappear or have limited expansion and will ultimately be outcompeted by wild‐type (nonmutant) hepatocytes and disappear over time. Another possibility is a mutant without a change to its survival probability as compared to the original hepatocyte, in which case the mutant cell will expand clonally at the same rate as wild‐type hepatocytes turn over/expand. Lastly, a mutation may result in a survival advantage to the cell, in which case the mutant cell population will expand more quickly than wild‐type hepatocytes. In these later two scenarios, if the mutation moves the cell further along the continuum towards malignant transformation, the odds of a similar subsequent event increase as the clone expands, increasing the probability that a second mutation or chromosomal translocation can occur, further increasing the risk for cancer. Red indicates cellular mutation. Yellow indicates cell death. Orange represents a red cell with an additional mutation that further improves survival. HBV, hepatitis B virus; HBx, hepatitis viral protein; HCC, hepatocellular carcinoma.

Moreover, HBV integrations (present in 80% of HCC tumours [66]) can facilitate chromosomal translocations that drive genomic instability and potentiate some types of malignant transformation (Figure 2). As far back as the 1980s, postmortem biopsy samples revealed substantial levels of integration of HBV DNA in both tumour and surrounding nontumor tissue [67, 68]. Integrations occur at sites of host‐cell double‐stranded DNA breaks via nonhomologous end joining [69, 70]. Viral integrations have been identified in both tumour and nontumor tissue (76.9% and 37.6%, respectively, in one study) [63, 71], and most HCC tumours have multiple integrations (both complete viral genomes and genomic fragments) in their chromosomes [11, 63, 72, 73, 74]. The number of tumour integrations is also correlated with patient mortality [12]. Recent studies using more sensitive techniques have shown that HBV DNA sequences are integrated into the host genome and facilitate chromosomal translocations throughout infection and do so with some frequency [14, 20], a likely early‐stage mechanism driving hepatocyte transformation [7]. Translocations have been shown to be present in both tumour and nontumor tissue (likely via bridge‐mediated chromosomal translocations) [75, 76] and are associated with an increased risk of genetic instability, which generates cells that have aberrant, missing, or extra chromosomes or contain chromosomes with altered gene copy numbers [77]. Such cells may include precancerous and cancerous cells [78].

HBV DNA insertional mutagenesis may result in the dysfunction of cancer‐associated genes (e.g., *telomerase reverse transcriptase* [*TERT*]). The *TERT* promoter regulates telomerase activity, which is highly associated with cancer risk [79]. In HBV‐related HCC, it has been shown that HBV DNA integrations frequently are found in *TERT* in tumour tissue from HBV patients (36% amongst a study of 95 patients with HCC) [80, 81]. Although *TERT* is associated with carcinogenesis, *TERT* promoter mutations alone do not result in tumorigenesis, as they have also been detected in precancerous histological lesions [82]. For patients with HCC (including those with HBV‐associated HCC), the average proportion of *TERT* integration sites was shown to be higher than that of other integration sites [83]. HBV integrations have also been found to alter other cancer‐associated genes, including lysine methyltransferase 2B (*KMT2B*) and Cyclin E1 (*CCNE1*) [84]. It is unclear whether integration is nonrandom, selecting certain genes as targets, or random, possibly generating lethal mutations in hepatocytes that are thus never seen.

An additional aspect of the chromosomal instability associated with HCC development and progression is the gain or loss of the region‐specific genomic DNA copy number; transcriptional levels of several important genes with genomic DNA copy number alterations (CNA) are deregulated in tissue from patients with HCC [85]. The chr1q and chr8p regions in particular are sites of frequent genomic amplifications and deletions, respectively, with amplified YY1AP1 (chr1q22) correlated between CNA and gene expression and CNA positively correlated with tumour grade [86]. In the analysis of tumour and nontumor tissue from 177 patients, 179 events (36%) of clonal integrations matched CNA boundaries in the human genome, indicating the relationship between viral insertion events and chromosome structural rearrangements [12]. These CNA‐associated integrations are clonally selected and appear to drive the development of cancer. Repeatedly observed types of CNA bordered by HBV integrations included large deletions of chromosome 17p, including TP53, which were often associated with centromeric insertions [12]; large gains at chromosome 8q, including MYC; and focal gains of *TERT* at 5p [12, 87].

Importantly, HBV DNA integration occurs within days of being infected with the virus, suggesting that pro‐oncogenic genetic lesions can occur early in an infection [21, 88]. One study of HBeAg‐negative patients demonstrated that genetic alterations potentially associated with carcinogenesis were associated with high viremia (> 20 000 IU/mL): 56% of patients vs. 14% and 25% of patients, respectively, with moderate and low viremia [76]. Moreover, the number of integrations correlated with viral load, and, importantly, transcriptionally active viral integrations were shown to be reduced by antiviral treatment [76, 89, 90]. The opportunity to prevent integrations in patients without transaminasemia underlines a likely benefit to early treatment in the form of cancer prevention [91]. Since these translocations are mediated by HBV integrations, and treatment ameliorates these, it is logical to assume treatment will reduce the incidence of translocations as well.

### 2.3 Chronic Inflammation and Clonal Expansion

It is common to see multiple copies of cells with the same integration patterns, a sign that they have clonally expanded (see Figure 2), a necessary condition of precancerous growth. Cells containing a single pro‐oncogenic change are unlikely to result in cancer [92]. Rather, an accumulation of changes in cell dysregulation/differentiation can cause transformation. The likelihood of this type of accumulation increases as a cell containing one or two premalignant alterations undergoes clonal expansion, increasing the chance that one out of this expanded population of cells acquires subsequent mutations sufficient to cause cancer. Additionally, the concomitant increase in the likelihood of the activation of the inflammatory cascade due to ongoing viral replication adds to this risk.

This occurs via two possible mechanisms: (1) by promoting pro‐oncogenic cell growth (e.g., through activation of cell cycling) and (2) cells escaping immune‐mediated clearance. Both of these increase the probability of clonal expansion of cells containing selective advantages (which can include oncogenic alterations) [93, 94, 95]. Clonal expansion followed by additional cellular changes in cell regulation/differentiation leads to an accumulation of precancerous cell populations that predispose to malignant transformation and tumour development.

Inflammation (caused by either HBV‐specific and non‐specific immune responses) increases the turnover of hepatocytes in the liver, thereby accelerating this process and allowing cells with heritable selective advantages to further flourish [9]. By limiting inflammation, treatment with antivirals can decrease the rate of clonal expansion and therefore may drive treatment‐associated reduction in cancer risk [90].

### 2.4 Changes in the Liver Microenvironment

Chronic inflammation and the concomitant cell cycle activation that is triggered in tissues that are experiencing heightened turnover create an environment with the potential to foster malignant transformation in itself. This is clearly indicated by the transformational events seen in nonalcoholic steatohepatitis and hepatitis C virus infection, as well as other inflammatory liver disorders that have no direct mutagenic effects [96, 97]. Like these other causes of hepatitis, hepatic inflammation observed with HBV has been associated with fibrosis, metaplasia, and tumorigenesis [9, 98], although neither inflammation nor fibrosis are a necessary prerequisite for liver cancer in HBV‐infected patients. Nonresolving hepatic inflammation can induce genomic instability, promote the development of liver cancer cells from liver progenitor cells [99], promote cell proliferation and survival, and activate tissue invasion [98]. Genes related to the JNK/c‐Jun pathway have tumour‐suppressing roles in liver carcinogenesis [100], such as influencing myeloid cell function [101]. Immunohistochemical testing found p‐JNK, c‐Jun, JunD, and AP‐1 to be present in 70%, 72.5%, 80%, and 62.5% of HCC cells [102]; while in hepatocytes these genes may show tumour‐suppressive effects, in nonparenchymal cells, they also contribute to an inflammatory environment that supports HCC development [103]. Hypoxia (driven by inflammation and subsequent changes in the microstructure of the liver) can produce cell necrosis, which in turn releases damage‐associated molecular pattern proteins. These proteins trigger downstream proinflammatory cascades, which contribute to HCC tumour progression [104]. Moreover, chronic inflammation and cellular turnover can be self‐perpetuating: chronic inflammation can lead to the establishment of fibrosis and cirrhosis, which alters blood flow and induces further cell death and inflammation in regenerative nodules [98, 105].

Transaminasemia (specifically, several somewhat arbitrary abnormal levels of ALT) is used as a marker of liver inflammation and an indication for antiviral treatment by several major guidelines [4, 7, 22, 23, 24]. However, the correlation between elevated ALT and inflammation in patients with HBV is imperfect, as 30% of patients with normal ALT show significant inflammation, and 24% of patients with elevated ALT and no significant fibrosis may not have significant inflammation [17]. The liver may have constant benign low‐level inflammation from a number of contributing factors; moreover, the likely mechanism by which blood levels of ALT rise is probably primarily due to necrotic cell death and less a result of apoptotic and necroptic cell death [106, 107]. A better marker that correlates with the breadth of inflammation‐induced cell death events in the liver remains to be found. Thus, treatment criteria not dependent on elevated aminotransferases should be strongly considered.

## 3 Current Treatment to Prevent HBV‐Associated HCC

Over the last few decades, antiviral medications have demonstrated the ability to reduce HBV viral load with an excellent safety profile. Beginning with the 1998 approval of the reverse transcriptase inhibitor lamivudine [108], a number of antivirals have become available, including adefovir dipivoxil [109], entecavir [110], telbivudine [109], and tenofovir (either as tenofovir disoproxil fumarate [TDF] or tenofovir alafenamide [TAF]) [4]. For a number of years, the two oral antivirals recommended as first‐line treatment, along with pegylated interferon (Peg‐IFN) for HBV, have been entecavir and TAF/TDF forms of tenofovir for their combination of potency in suppression of viral replication and their outstanding resistance profiles [4, 7].

In addition to lowering HBV viral load, studies have shown that antiviral therapy can decrease the likelihood of developing HCC [111]. A retrospective‐prospective cohort study in Hong Kong studied entecavir in patients with CHB infection and cirrhosis. Only patients who maintained viral suppression had a significantly lower probability of HCC compared to untreated control cohorts. Entecavir also resulted in a lower probability of hepatic‐related adverse events (AEs) and mortality amongst patients with cirrhosis [112]. Similar findings in lowering HCC risk were also reported in CHB patients receiving entecavir followed for over 3 years [113]. Amongst patients treated with oral antiviral therapy, HCC occurred less frequently in those with complete vs. incomplete viral suppression [114]. Repeated studies have further demonstrated the ability of antiviral treatment to prevent HCC, and several are summarised in Table 3. Additional corroboration comes from HBV/HIV‐coinfected patients, who, unlike immune‐tolerant patients with HBV monoinfection, are almost universally prescribed antiviral treatment and show a substantially lower rate of HCC [115, 116].

Treatment is recommended by societal guidelines for those with certain cutoffs for viral load (> 2000 IU/mL in HBeAg‐negative patients and > 20 000 IU/mL in HBeAg‐positive patients), stipulated degrees of elevation in ALT (ideally, 2× upper limit of normal [ULN]) purportedly corresponding to active disease (i.e., “immune control” and “reactivation” phases), or those with a family history of liver cancer [4, 7]. Recent changes to the EASL guidelines have also included considering anyone older than 30 years with high viral load for treatment, though these are still not outright recommendations (Table 2). In contrast, the AASLD suggests antiviral therapy in the select group of adults > 40 years of age with normal ALT and elevated HBV DNA (1 000 000 IU/mL) and liver biopsy showing “significant necroinflammation or fibrosis” [4].

### 3.1 HCC Screening and Diagnosis

AASLD guidelines advise that HBV‐infected patients with cirrhosis as well as noncirrhotic patients meeting certain demographic criteria (age, gender, ethnicity, family history) undergo an abdominal ultrasound every 6 months to detect development of HCC (Table 4) [4, 117], and EASL guidelines recommend that all patients with CHB infection undergo an abdominal ultrasound at their initial assessment [7]. However, ultrasound has low sensitivity at early stages of HCC [118] and can be influenced by factors related to the operator and patient, such as operator skill, interoperator variability, and differences in body habitus [119, 120]. Cross‐sectional imaging is more sensitive for small HCCs but more costly and not recommended as a first‐line screening procedure [121]. Nevertheless, many clinicians obtain an MRI when available, at least at periodic intervals, and especially when technical factors limit the sensitivity of ultrasound, including the coarse or heterogeneous echotexture and nodularity that reduce the sensitivity of ultrasound. Aside from imaging, serum biomarkers such as AFP are used to diagnose HCC, although AFP elevations are found in only 60%–70% of patients with HCC [117, 122]. More research into the utility of such markers as hepatitis B core‐related antigen (HBcrAg) and pre‐S mutation biomarkers is needed, particularly in terms of practicability in clinical settings [119]. Future studies should identify biomarkers, potentially including liquid biopsy, that can enhance early detection of HCC.

**TABLE 4:** Groups who are recommended to be screened for HBV in the US.

Groups recommended for HBV screening		
Related to vertical transmission	Related to horizontal transmission	Comorbidities
Persons born in regions of high or intermediate HBV endemicity (HBsAg prevalence of > 2%)	Healthcare and public safety workers at risk for occupational exposure to blood or blood‐contaminated body fluids^a^	Persons with HIV^a^
US‐born persons not vaccinated as an infant whose parents were born in regions with high HBV endemicity (≥ 8%)^a^	Residents and staff of facilities for developmentally impaired persons^a^	Persons undergoing immunosuppressive therapy (e.g., chemotherapy, organ transplantation, rheumatologic or gastroenterologic disorders)
All pregnant women	Persons who are exposed to blood or other bodily fluids that might require postexposure prophylaxis	Persons with chronic liver disease^a^
Infants born to HBsAg‐positive mothers^a^	Inmates of correctional facilities^a^	Unvaccinated persons with diabetes aged 19–59 years (discretion of clinician for unvaccinated adults with diabetes who are aged ≥ 60)^a^
	Travellers to countries with intermediate or high prevalence of HBV infection^a^	Persons with end‐stage renal disease, including predialysis, haemodialysis, peritoneal dialysis, and home dialysis patients^a^
	Men who have sex with men^a^	Individuals with elevated ALT or AST of unknown etiology^a^
	Persons who are not in long‐term, mutually monogamous relationships (e.g., > 1 sex partner during the previous 6 months)^a^	Persons seeking evaluation or treatment for a sexually transmitted disease^a^
	Persons who have ever injected drugs^a^	Donors of blood, plasma, organs, tissues, or semen
	Household of, needle sharing with, or sexual contacts of HBsAg‐positive persons^a^	

*Note:* Adapted from AASLD Hepatitis B Guidance.

Abbreviations: AASLD, American Association for the Study of Liver Diseases; ALT, alanine aminotransferase; AST, aspartate aminotransferase; HBsAg, hepatitis B surface antigen; HBV, hepatitis B virus; HIV, human immunodeficiency virus.

^a^ Indicates those who should receive the HBV vaccine, if seronegative.

## 4 The Case for Expanding the Populations Considered for Treatment

Several studies have suggested that CHB patients who do not meet criteria for treatment in professional society guidelines or are at an “indeterminate” phase are at risk for HCC that may be preventable with antiviral therapy. In a study of 3624 HBV patients, of whom 161 developed incident HCC, APASL, AASLD, or EASL guideline‐specified criteria for treatment were unmet by 64%, 46%, and 33.5% [123]. A recent study involving 14 international centers evaluated 855 CHB patients without advanced fibrosis in the “indeterminate phase.” Inverse probability of treatment weighting was used to balance treated and untreated patients. The cumulative incidence of HCC was 3% in both groups at 3 years, but 15% and 19% at 10 and 15 years, respectively, in the untreated patients vs. 4% and 9% in the treated patients at 10 and 15 years, respectively [124]. Recent changes to World Health Organization guidelines as well as guidelines in China [22] and recent changes in the US reflected by the US Treatment Algorithm [23], a recent Simplified Treatment Algorithm (SABA) [125], and another recent proposed algorithm [126] will encompass a real‐world cohort of patients that can be expected to demonstrate improved outcomes over time. The 2023 Chinese Guidelines are particularly notable for their expansiveness, recommending antiviral therapy for *any* detectable HBV DNA and ALT > ULN or, if over 30 years old, ALT < ULN (or family history of HCC or significant inflammation and fibrosis) – although still not recommending antiviral therapy for patients under 30 years of age with normal ALT but very high viral levels [127]. The recent World Health Organization Guidelines removed HBV DNA completely from three out of four categories of patients considered to warrant therapy, doubtless reflecting concerns about the lack of accessibility of HBV DNA testing in less developed countries [128]. From a practical viewpoint, randomised controlled trials of sufficient duration and numbers of patients cannot be realistically expected in these expanded populations. In the interim, many patients globally may experience preventable cancers before the benefits of these newer recommendations are realised, and the scope of even the newest guidelines does not include all patients who will later develop HCC. We propose a broader global expansion of treatment consideration. Several arguments exist for expanding treatment to address the impacts of CHB infections on people living with HBV, including limiting inflammation (and subsequent liver disease progression) in patients currently outside treatment guidelines, reducing cccDNA levels to potentially increase the efficacy of curative treatments [129, 130], preventing additional procarcinogenic effects (integrations and clonal expansion) [131], empowering patients to prevent transmission themselves without having to refer intimate contacts for vaccinations that they may not have access to or may not respond to, and, through all these, improving the quality of life of people living with HBV [132].

### 4.1 Limiting Inflammation

ALT levels are commonly used to evaluate patients with HBV, and antiviral treatment is often not begun until ALT levels are elevated to arbitrary levels that are considered abnormal. For patients with HBV DNA > 2000 IU/mL, ALT > ULN (approximately 40 IU/L), and moderate liver necroinflammation or fibrosis, guidelines from EASL direct that treatment should be initiated in both HBeAg‐positive and ‐negative patients. Both AASLD and EASL guidelines state that patients with HBV DNA > 20 000 IU/mL and ALT > 2× ULN should start treatment (EASL recommends treatment regardless of the degree of fibrosis, while AASLD recommends it in cases of moderate/severe inflammation or significant fibrosis) [4, 7]. While most guidelines recommend starting antiviral therapy when both HBV DNA and ALT levels are elevated, viral integration is already underway during the immune‐tolerant (HBeAg‐positive CHB infection) phase, when ALT levels may be normal or only slightly elevated [21, 88].

Recent studies demonstrate that there is activation of various components of the inflammatory cascade even during the immune‐tolerant phase of infection [133]. Inflammation not only drives liver disease but also represents a route towards malignant transformation. Since it is believed immune activation is primarily driven by *de novo* infection of uninfected hepatocytes (a mechanism that explains why treatment reduces inflammation) [134], this represents a reason to consider earlier treatment initiation.

### 4.2 cccDNA Reduction

Wong et al. reported a 1.0 log reduction in intrahepatic cccDNA amongst 39 patients receiving NA therapy for 48 weeks, with no significant difference in change between entecavir and lamivudine [130]. Recent research by Chow et al. showed a 39% reduction in integrations and a 1.0 log reduction in cccDNA after 1 year of antiviral treatment; a subgroup of patients with 10 years of treatment showed an 88% reduction in integrations and a 2.3 log reduction in cccDNA; patients also had significant reductions in serum HBV DNA but not in HBsAg [90].

### 4.3 Preventing the Establishment of Cancer Drivers

A historical cohort study in Korea showed that HBV‐infected persons who were in the immune‐tolerant phase and untreated had a higher incidence of HCC and death compared with immune‐active patients receiving antiviral therapy, suggesting that earlier antiviral intervention may have led to better outcomes [30].

One likely driver of cancer during this period is HBV DNA integration. Any HBV replication generates dslDNA‐containing virions that can infect cells, integrate into the host genome, and start a cell down the road to malignant transformation [135]. Once established, there is no known way to eliminate mutant populations of cells. However, earlier treatment with nucleoside antivirals could prevent new integrations from occurring and limit the inflammation that drives clonal expansion, in theory preventing liver cancer.

Indeed, long‐term NA administration reduces the number of integrations, the number of clonally expanded populations of cells, and the number of transcriptionally active viral integrations [90, 136]. Over a 3‐year period in a recent study, there was a statistically significant greater reduction in the number of integrations in patients treated with tenofovir vs. placebo [136]. We posit that this explains the clinical observations that demonstrate that antiviral treatment mitigates HBV‐associated cancer risk [14, 137]. Though current guidelines recommend only monitoring patients in the immune‐tolerant phase, we believe strong consideration should be given to informing these patients of the benefits of treatment and offering it when requested [4].

### 4.4 Improving the Lived Experience With CHB

Since these mutations occur throughout HBV infection, it seems important and fitting that patients are made aware of the possible risks and consequences of virally induced mutagenesis and its relationship to the development of cancer as well as the role of NA treatment in mitigating this risk and stopping the process of mutagenesis. In this way, patients are empowered to make informed decisions regarding their individual comfort levels with exposure to a mutagen. In addition, since patients that have no virus in their body fluids due to antiviral treatment are less likely to transmit infection to others, it empowers them to have control of their disease and relieves the burden of worrying about their risk to others. Indeed, it is not unusual for young people with CHB to self‐ostracise and limit potential relationships because of these concerns [138].

## 5 Barriers to the Expansion of Treatment Criteria

### 5.1 Difficulties in Showing Anti‐HCC Effect Through Randomised Clinical Trials

Prospective randomised controlled trials—or observational data resulting from the new Chinese or simplified guidelines—are unlikely to be imminently forthcoming to illustrate the impact of early treatment on carcinogenesis given the long period between infection, treatment, and HCC development. The REVEAL study showed that the probability of developing HCC amongst HBeAg‐negative HBV patients with normal ALT and serum levels of HBV DNA < 10 000 copies/mL was less than 1% after 13 years of follow‐up [10]. This is similar to what is seen with lung cancer development for former pack‐a‐day smokers when followed for a similar length of time [139]. It is important to recognise that this is not evidence that hepatocarcinogenesis is not taking place so much as it is substantiation that the changes that lead to malignant transformation typically occur over decades, not years.

In these populations with low (but still above normal) HCC rates, studies would be logistically and financially impractical, as they would have to be carried out on very large populations with decades‐long follow‐up to show effect. The lack of a small‐animal model that recapitulates the carcinogenic process observed in human HBV infections also limits the hope of testing these concepts; in the available woodchuck model, HCC develops a median 2–2.5 years after neonatal infection with the analogous hepatitis virus, virions from which are present in concentrations 10‐ to 100‐fold greater than in humans with CHB [140]. Nevertheless, the numerous advantages (including likely direct health benefits) associated with broadened antiviral therapy criteria should be seriously considered, and, regardless of actual incidence, the risks vs. benefits associated with treatment should be discussed with patients.

### 5.2 Risks of Treatment

As summarised in the most recent publication of the US Treatment Algorithm, most experts have traditionally felt that universal treatment is not warranted because immune‐tolerant patients were perceived as being at little risk and out of concerns about the possibility of treatment being necessary for decades [23]. It is important that patients be educated on the reality of risks in order to best gauge the potential benefits of treatment. There are 3 first‐line drugs available for treatment in most of the developed world. Two of the 3 (ETV and TDF) are generic in most countries. Studies in many countries have demonstrated that universal treatment of HBV is cost effective [141, 142, 143]. Informed patient choice is critical to presenting and discussing treatment options. The decision to commit to protracted therapy may be difficult or undesirable for many patients, especially as most have little symptomatology [144]. Yet it is essential that patients be informed of the risks and consequences of deferring treatment, including a distinct likelihood of durable and potentially irreversible changes to hepatocytes, which are likely occurring whenever HBV is replicating.

Given that some patients may be “overly compliant” and accept physicians' recommended treatment plans regardless of cost or collateral consequences [145], expansion of treatment criteria must be done with care to ensure that patient choice is well informed and based on evidence. Individual patients' circumstances and HBV disease courses are unique, and open communication regarding risks when planning disease management should lead to better outcomes.

The impact of AEs associated with long‐term NA treatment on patient quality of life has been posited as an argument against expansion of early treatment. The most common AEs associated with NA use include typically mild‐to‐moderate headache and fatigue [146]. More serious AEs are rare but include renal toxicity and self‐limited mild changes in bone mineral density (for TDF) [147]. The risk of emergent resistance is also often invoked in this context but is rare with entecavir (1% in patients not previously treated with lamivudine) and has not been reported with tenofovir in over 10 years of follow‐up [7]. Many people may be willing to forgo these risks in favour of the benefits when presented with an informed discussion of the considerations outlined here. For example, HIV‐negative people with HIV‐positive partners sometimes take some of the same antivirals used in HBV treatment for pre‐exposure prophylaxis, which has been recommended by the World Health Organization since 2014 [148].

Universal treatment of HBV has been demonstrated to be cost‐effective in multiple countries [141, 142, 143], and resistance to at least 2 of the first‐line therapies has never been seen; these also have safety profiles supporting use in healthy, virus‐free individuals for HIV prevention. We believe the case for offering NA therapy to prevent cancer is strong.

## 6 Conclusions

One and a half million new infections and more than 800 000 deaths continue to occur each year due to CHB infection [1], despite the firmly established link to liver cancer, a highly efficacious vaccine, and safe, off‐patent, orally available drugs to prevent HBV disease progression. Treatment and diagnosis rates for HBV have been stagnant at current levels for decades. The tools available to the medical community must be used much more effectively to have an impact on these appalling morbidity and mortality rates; the status quo is not working and does not reflect the urgency needed to stem morbidity and mortality. Ambitious measures in screening and expansion of treatment need to be considered to properly address the carcinogenic threat faced by 300 million people worldwide.

## Conflicts of Interest

T.T. reports receiving grant funding from Excision Biosciences and Gilead Sciences Inc.; is president of the Australian Centre for Hepatitis Virology; is founder/director of HepBcommunity.org; and reports being a consultant for Excision Biosciences; Gilead Sciences Inc.; and GSK. T.J.M. reports being an employee of Gilead Sciences Inc., and may own stock. I.M.J. reports being a consultant or on advisory boards for AbbVie; Aligos Therapeutics; Arbutus Biopharma; Cymabay; Gilead Sciences Inc.; Intercept; Janssen; Madrigal; Merck; and Moderna; having conducted research (all payments to institution) for Assembly Biosciences; AusperBio; Bristol Myers Squibb; Cymabay; Eli Lilly; Enanta Pharmaceuticals; Gilead Sciences Inc.; GSK; Ipsen; Janssen; Merck; Mirum; Novo Nordisk; Rockefeller University (NIH funded); receiving payment from the Chronic Liver Disease Foundation for manuscript preparation; and participation on a Data Safety Monitoring Committee for Aligos Therapeutics, Altimmune, GSK, and Takeda.

## Data Availability

The data that support the findings of this study are available from the corresponding author upon reasonable request.
